# Complications During the Hospital Stay, Length of Stay, and Cost of Care in Parkinson Patients Undergoing Total Knee Arthroplasty: A Propensity Matched Database Study

**DOI:** 10.5435/JAAOSGlobal-D-22-00121

**Published:** 2022-07-08

**Authors:** Naga Suresh Cheppalli, Tejas Senthil, Vishaal Sakthivelnathan, Anil Menedal, Varatharaj Mounasamy, Senthil Sambandam

**Affiliations:** From the University of New Mexico School of Medicine (Dr. Cheppalli); the Staff orthopedic Surgeon, Raymond Murphy Medical Centre, VAMC, Albuquerque, NM (Dr. Cheppalli); the Carroll High School, Southlake, TX; the School of Medicine, University of Texas medical Branch, TX (Mr.Sakthivelnathan); the Staff Orthopedic Surgeon, Salem VA Medical Center Salem, Virginia (Dr.Menedal); the Department of Orthopedics (Dr. Mounasamy), Dallas VAMC; and the University of Texas Southwestern (Dr. Sambandam), Staff Orthopedic Surgeon, Dallas VAMC, Dallas, TX.

## Abstract

Patients with Parkinson disease (PD) undergoing total knee arthroplasty (TKA) can present with a unique subset of challenges during their hospital stay. The literature is limited to single-center studies with a small number of patients. This study was aimed to analyze the inpatient complications, length of stay (LOS), and cost of care (COC) associated after TKA with PD over 4 years (2016 to 2019).

**Methods:**

In this retrospective cohort study, we used National Inpatient Sample (NIS) database data from 2016 to 2019 and compared in-hospital complications, LOS, and COC among patients undergoing TKA with and without PD.

**Results:**

The National Inpatient Sample database is used to identify 558,371 patients (555,289 without PD and 3,082 with PD) who underwent TKA. After propensity-matching, there was an increased incidence of blood loss anemia (PD group 22.3%, control group 13.5%, *P* ≤ 0.01), periprosthetic dislocations (1.5% in PD group, 0.4% in control group, *P* < 0.001), and periprosthetic mechanical complications including but not limited to periprosthetic fractures, knee dislocations, patellar maltracking, and subluxations (1.1% in PD group, 0.6% in control group, *P* = 0.024) in the PD group. The other in-hospital complications including mortality, acute renal failure, myocardial infarction, pneumonia, pulmonary embolism, deep vein thrombosis, periprosthetic fracture, and wound dehiscence showed no notable differences. The average total incurred charges for the PD group were higher, with a mean of $67,581.58 (SD $44,554.64), than that in the control group, with a mean of $64,795.51 (SD $45,841.25) (*P* < 0.001). The average LOS was higher in the PD group with a mean of 2.7 days (SD = 1.93) compared with the control group (mean = 2.3, SD = 1.73 days, *P* < 0.001).

**Conclusions:**

An increased incidence of complications such as acute blood loss anemia, periprosthetic mechanical complications, and increased COC, but no difference in LOS was noted in patients undergoing TKA with PD. This information can be useful to make an informed decision regarding patient care and preferred healthcare setup for TKA in patients with PD.

The incidence of Parkinson disease (PD) among patients undergoing total knee arthroplasty (TKA) is 0.056%.^1^ In an estimate, there will be 1,238,000 patients with PD by 2030,^2^ and the incidence of TKA is expected to increase 143% by 2050.^3^ With these statistics, it is anticipated that an increased number of patients with PD will be undergoing TKA in the near future. PD is a progressive neurodegenerative disease with decreased dopaminergic activity in the nigrostriatal pathway leading to a dysfunction of the basal ganglia. It is characterized by motor disturbances and cognitive impairment with multiple stages (modified Hoehn and Yahr staging and Columbia staging).^4^ This disease is primarily characterized by tremor, bradykinesia, dystonia, postural changes, loss of balance, dizziness, and drooling and can involve other systems as well. Secondary to systematic involvement of the disease, it is anticipated that there will be an increased incidence of immediate postoperative complications, thus leading to increased length of stay (LOS) and cost of care (COC). In the era of bundled payment and outpatient TKA, this information can be useful for orthopaedic surgeons to make an informed decision regarding patient care and the place of patient care (hospital-based versus surgery center) and negotiate a contract with the payers. This study aimed to analyze the inpatient complications associated after TKA with PD over 4 years (2016 to 2019) using the National Inpatient Sample (NIS) database.

## Methods

### Database Description

This study was completed using the NIS database data from 2016 to 2019. The NIS database is a component of the Healthcare Cost and Utilization Project. It is the largest all-payer inpatient care database within the United States, with data from more than 7 million hospital stays per year. The data encompass 20% of the hospitals in the United States and are verified using a quality assessment evaluation comparing data points with standardized normative values by an independent contractor.

Data elements within the NIS database include demographic data, LOS, payment source, hospital charges, discharge status, comorbidities, and more. The 2016 to 2019 version uses the International Classification of Diseases (ICDs), Tenth Revision, Clinical Modification/Procedure Coding System.

### Data Acquisition

This study was exempt from IRB approval because the data were deidentified and publicly available. Patients who underwent TKA were identified using the ICD-10 procedural codes OSRC and OSRD. Data from patients with PD were extracted using the ICD code G20 (Table 1).

**Table 1:** International Classification of Disease Codes Used

Parkinson Disease	Obese Codes	Morbidly Obese codes	Comorbidities codes	Medical Complications codes	Surgical Complications codes
G20	E660 E6601 E6609 E661 E662 E668 E669 Z6830 Z6831 Z6832 Z6833 Z6834 Z6835 Z6836 Z6837 Z6838 Z6839	Z6841 Z6842 Z6843 Z6844 Z6845	Diabetes without complications E119 Diabetes with complications E1169 Tobacco-related disorder Z87891	Acute renal failure N170, N171, N172, N178, and N179 Myocardial infarction I2101, I2102, I2111, I2113, I12114, I12119, I2121, I12129, and I21A1 Blood loss anemia D62 Pneumonia J189, J159, and J22 Blood transfusion 30233N1 Pulmonary embolism I2602, I2609, I2692, and I2699 DVT I82401,I82402, I82403, I82409, I82411, I82412, I82413, I82419, I82421, I82422, I82423, I82429, I82431, I82432, I82433, I82439, I82441, I82442, I82443, I82449, I82491, I82492, I82493, I82499, I824Y1, I824Y2, I824Y3, I824Y9, I824Z1, I824Z2, I824Z3, and I824Z4	Periprosthetic fracture T84010A, T84011A, T84012A, T84013A, T84018A, T84019A, M9665, M96661, M96662, M96669, M96671, M96672, M96679, M9669, M9701XA, M9702XA, M9711XA, and M9712XA Periprosthetic dislocation T84020A, T84021A, T84022A, T84023A, T84028A, and T84029A Periprosthetic mechanical complications T84090A, T84091A, T84092A, T84093A, T84098A, and T84099A Periprosthetic infection T8450XA, T8451XA, T8452XA, T8453XA, T8454XA, and T8459XA Superficial SSI T8141XA Deep SSI T8142XA Wound dehiscence T8130XA, T8131XA, and T8132XA

Preoperative variables included (1) age, (2) sex, (3) elective versus nonelective admission, (4) diabetes without complications, (5) tobacco use disorder, and (6) obesity (Table 2). Postoperative medical and surgical outcomes aggregated from the NIS database include (1) LOS, (2) total incurred charges, (3) mortality, (4) elective versus nonelective admission, (5) acute renal failure, (6) myocardial infarction, (7) blood loss anemia, (8) pneumonia, (9) pulmonary embolism, (10) deep vein thrombosis, (11) periprosthetic fracture, (12) periprosthetic dislocation, (13) periprosthetic mechanical complication, (14) periprosthetic infection, (15) superficial surgical site infection, (16) deep surgical site infection, (17) wound dehiscence, (18) cardiac arrest and ventricular fibrillation, and (19) blood transfusion (Table 3). These complications are representative of the most common complications of TKA available on the NIS database.

**Table 2:** Patient Demographics

	PD Group (3082)	Control Group (555289)	Odds Ratio (PD Group/Control Group)	Odds Ratio 95% Confidence Interval	Significance
Mean age (SD) in years	71.44 (7.882)	66.59 (9.507)	NA	NA	*P* < 0.001
Sex (proportion of female)	1549 (50.3%)	341871 (61.66)	0.631	[0.588, 0.677]	*P* < 0.001
Diabetes without complication (proportion of diabetic)	376 (12.2%)	82008 (14.8%)	0.801	[0.719, 0.892]	*P* <0.001
Tobacco use disorder (proportion of users)	356 (11.6%)	88013 (15.8%)	0.693	[0.621, 0.775]	*P* <0.001
Obesity (proportion of obese)	643 (20.9%)	172018 (31.0%)	0.587	[0.538, 0.641]	*P* <0.001

**Table 3:** Unmatched Analysis

Postoperative Variables	PD Group (3082)	Control Group (555289)	Odds Ratio (PD Group/Control Group)	Odds Ratio 95% Confidence Interval	Significance
Mortality	0	198 (0.04%)	NA	NA	0.294
Acute renal failure	67 (2.2%)	11018 (2.0%)	1.098	0.861-1.399	0.451
Myocardial infarction	0	109 (0.02%)	NA	NA	0.437
Blood loss anemia	587 (19.0%)	84970 (15.3%)	1.302	1.19-1.425	<0.001
Pneumonia	12 (0.4%)	1076 (0.2%)	2.013	1.139-3.56	0.014
Pulmonary embolism	^b^0.2%	1231 (0.2%)	1.025	0.487-2.156	0.949
Deep vein thrombosis	11 (0.4%)	1251 (0.2%)	1.586	0.875-2.875	0.125
Periprosthetic fracture	18 (0.6%)	2345 (0.4%)	1.385	0.870-2.206	0.168
Periprosthetic dislocation	47 (1.5%)	4215 (0.8%)	2.025	1.515-2.705	<0.001
Periprosthetic mechanical complication	34 (1.1%)	4470 (0.8%)	1.375	0.979-1.930	0.065
Periprosthetic infection	38 (1.2%)	5764 (1.0%)	1.190	0.863-1.641	0.287
Wound dehiscence	^a^0.2%	525 (0.09%)	1.717	0.711-4.146	0.224
Blood transfusion	68 (2.2%)	8186 (1.5%)	1.508	1.185-1.919	<0.001

a The exact number is not reported if the value is between 1 to 10 as per the Healthcare Cost and Utilization Project data use agreement.

### Statistical Analysis

All statistical analyses were conducted using SPSS version 27.0 (IBM). Originally, descriptive statistics were used to aggregate patient demographic data. An unmatched analysis and matched analyses were completed. A 1:1 propensity-matching algorithm using the preoperative variables was performed. Student *t*-tests were used when analyzing numerical variables. Chi square analyses were used when analyzing binomial variables. Fisher exact tests were used when the incidence values were less than 5. A *P* value of < 0.05 was considered statistically significant for all tests. Odds ratios and their corresponding 95% confidence intervals for the surgical outcomes and complications were measured as a ratio of the incidence in the control group to the incidence in the PD group.

## Results

A total of 558,371 patients who underwent TKA were identified using the NIS database. Within that population, 555,289 patients did not have PD and 3082 patients had PD (0.55%).

### Demographic Data

PD group patients were older (mean = 71.4 years) compared with the control group patients (66.59 years), and this difference is statistically significant (*P* < 0.001) (Table 2). The PD group tended to have fewer female patients, 50.3%, than the female patients in the control group, 61.6% (*P* < 0.001). The tobacco-related disorder incidence was less in the PD group (10.3%) than in the control group (15.8%, *P* = 0.032). The other demographic variables were not significantly different among the two groups (Table 1).

### Unmatched Postoperative Outcomes Analysis

The incidence of blood loss anemia was greater in the PD group, 19%, than in the control group, 15.3% (*P* < 0.001). The incidence of pneumonia was greater in the PD group, 0.3%, than in the control group, 0.1% (*P* = 0.014). The incidence of periprosthetic dislocation was greater in the PD group, 1.5%, than in the control group, 0.7% (*P* < 0.001). The incidence of blood transfusions was greater in the PD group, 3.9%, than in the control group, 1.5% (*P* = 0.004). The other postoperative outcomes showed no significant differences among the two groups in the unmatched analysis (Table 3).

The average total incurred charges for the PD group were higher, with a mean of $67,581.58 and a SD of $44,554.64, than that for the control group, with a mean of $64,795.51 and a SD of $45,841.25 (*P* < 0.001). The average LOS was higher in the PD group with a mean of 2.7 days and a SD of 1.933 days compared with the control group mean of 2.3 days and a SD of 1.730 days (*P* < 0.001) (Table 4).

**Table 4:** Length of Stay and Cost of Care in Unmatched and Matched Cohorts

	Unmatched PD	Unmatched Control	*P*	Matched PD	Matched Control	*P*
Length of stay	2.74 [1.730]	2.34 [1.933]	<0.001	2.74 [1.730]	2.94 [3.297]	0.003
Hospital charges	67581.58 [44554.640]	64795.51 [45841.245]	<0.001	67581.58 [44554.640]	46493.28 [71072.269]	<0.001

PD = Parkinson disease

### Matched Postoperative Outcomes Analysis

The 1:1 propensity-matched algorithm yielded 3,082 patients in the PD group and 2,967 patients in the control group (Table 5). The incidence of blood loss anemia was greater in the PD group, 22.3%, than in the control group, 13.5% (*P* ≤ 0.01). The incidence of periprosthetic dislocations was greater in the PD group, 1.5%, than in the control group, 0.4% (*P* < 0.001). The incidence of periprosthetic mechanical complication was greater in the PD group, 1.1%, than in the control group, 0.6% (*P* = 0.024). The other postoperative outcomes showed no significant differences among the two groups in the matched analysis (Table 6).

**Table 5:** Patient Demographics of the Matched Cohort

	PD Group (3082)	Control Group (2967)	Odds Ratio (PD Group/Control Group)	Odds Ratio 95% Confidence Interval	Significance
Mean age (SD) in years	71.44	71.04	NA	NA	0.074
Sex (proportion of female)	1549	1498	0.992	(0.896, 1.097)	0.869
Diabetes without complications (proportion of diabetic)	376 (12.2%)	362 (12.2%)	1.00	(0.857, 1.166)	0.999
Diabetes with complications	18 (0.6%)	17 (0.6%)	1.019	(0.524, 1.982)	0.955
Tobacco use disorder (proportion of users)	356 (11.6%)	339 (11.4%)	1.012	(0.864, 1.186)	0.879
Obesity (proportion of obese)	643 (20.9%)	621 (20.9%)	0.996	(0.880, 1.127)	0.949
Race White Black Hispanic Asian Native American	2543 (82.5%) 99 (3.2%) 206 (6.7%) 55 (1.8%) 16 (0.5%)	2541 (85.6%) 99 (3.3%) 206 (6.9%) 55 (1.9%) 16 (0.5%)	NA	NA	1.000
Age categorical <60 60-70 70-80 80-90 >90	222 (7.2%) 998 (32.4%) 1365 (44.3%) 483 (15.7%) 14 (0.5%)	213 (7.2%) 960 (32.4%) 1319 (44.5%) 462 (15.6%) 13 (0.4%)	NA	NA	1.000

PD = Parkinson disease

**Table 6:** Matched Sample Analysis

Post Operative Variables	PD Group (3082)	Control Group (2967)	Odds Ratio (PD Group/Control Group)	Odds Ratio 95% Confidence Interval	Significance
Mortality	0				
Acute renal failure	67 (2.2%)	62 (2.1%)	1.041	0.734-1.476	0.821
Myocardial infarction	0	^b^0.03%	0.490	0.478-0.603	0.308
Blood loss anemia	587 (19.0%)	402 (13.5%)	1.501	1.307-1.724	**<0.001**
Pneumonia	12 (0.4%)	11 (0.4%)	1.05	0.463-2.384	0.906
Pulmonary embolism	^b^0.2%	^b^0.2%	0.963	0.337-2.748	0.943
Deep vein thrombosis	11 (0.4%)	^b^0.3%	1.177	0.487-2.845	0.717
Periprosthetic fracture	18 (0.6%)	^b^0.3%	1.931	0.866-4.305	0.102
Periprosthetic dislocation	47 (1.5%)	12 (0.4%)	3.813	2.019-7.203	**<0.001**
Periprosthetic mechanical complication	34 (1.1%)	17 (0.6%)	1.936	1.079-3.472	**0.024**
Periprosthetic infection	38 (1.2%)	35 (1.2%)	1.046	0.659-1.660	0.849
Wound dehiscence^a^	(0.2%)	10 (0.3%)	0.481	0.164-1.407	0.172
Blood transfusion	68 (2.2%)	78 (2.6%)	0.836	0.601-1.161	0.284

PD = Parkinson disease

a The exact number is not reported if the value is between 1 to 10 as per the Healthcare Cost and Utilization Project data use agreement. Bold entries are statistically significant.

The average total incurred charges for the PD group were higher in the PD group, with a mean of $67,581.58 and a SD of $44,554.640, than in the control group, with a mean of $46,493.28 and a SD of $71,072.269 (*P* < 0.001). The average LOS was lower in the PD group, with a mean of 2.7 days and a SD of 1.730 days, than in the control group, with a mean of 2.9 days and a SD of 3.297 days (*P* = 0.101).

## Discussion

This study revealed that PD incidence among patients undergoing TKA is 0.55% and has not markedly changed since 2000 to 2012.^1^ We also observed that TKA with underlying PD is associated with an increased incidence of immediate postoperative complications such as acute blood loss anemia (ABLA), increased incidence of blood transfusion, increased COC, and increased knee prosthesis dislocation in the matched cohort analysis. Although we found statistically significant differences in LOS and the incidence of pneumonia in the unmatched cohort, we did not find a notable difference in the 1:1 matched cohort.

The total blood loss after TKA is estimated to be around 300 to 500 mL and is mostly hidden.^5^ However, several factors such as types of patient-related factors (age, if the patient is on anticoagulation medications), using tranexamic acid, anesthetic choice, tourniquet, surgical technique, prosthesis, and use of postoperative drain can influence the incidence of blood loss after TKA. In our study, we noted an increased incidence of ABLA (22.3%) in patients with PD when compared with the matched control group (13.5%) (*P* ≤ 0.01). Multiple medical and surgical factors related to PD can influence blood loss. It is hypothesized that medications used to treat PD can cause abnormalities in the coagulation-fibrinolysis process.^6^ However, several other factors such as surgical exposure, increased surgical time (required to overcome the exposure secondary to rigidity of hamstrings), and implant choice (more use of revision and constrained prosthesis to balance the knee joint and avoid dislocations causes more blood loss^7^) may have a notable influence on this outcome.

Patient LOS is frequently quoted as an outcome measure and compares between hospitals and surgeons. To strive in the competitive market, hospitals incorporated enhanced recovery pathways for the postoperative recovery period. Patients getting discharged in the morning leads to improved bed availability and lower cancellations in surgical patients, thereby highlighting the effect of even few hours of increased LOS.^8^ Bed availability became more apparent during the COVID-19 pandemic when there were restrictions for elective surgeries. In the unmatched cohort, the average LOS was higher in the PD group with a mean of 2.7 days and a SD of 1.9 days compared with the control group mean of 2.3 days and a SD of 1.7 days (*P* < 0.001). However, in a matched cohort, the average LOS was lower in the PD group, with a mean of 2.7 days and a SD of 1.7 days, than in the control group, with a mean of 2.9 days and a SD of 3.2 days (*P* = 0.101). An increase in LOS can be multifactorial, including medical and logistic factors. An increased incidence of postoperative confusion, urinary retention, and respiratory infection secondary to aspiration pneumonia, failure of the knee extensor mechanism, dislocation, and patellar subluxation are some of the reported medical reasons which can increase LOS. Rehabilitation of a patient with PD can involve multiple specialists and more trained personnel. The coordination of care between all the teams can itself delay the discharge. General anesthesia opiates can increase the incidence of postoperative confusion and can be avoided whenever possible in these subsets of people.^9^ Early neurological consultation can decrease LOS and improve outcomes. The stress of the surgery can aggravate the PD, and interventions such as dosing adjustments and narcotic management might help in reducing the LOS.^10^

Postoperative dislocations after TKA are rare and were previously reported in PD. The incidence of periprosthetic dislocations was greater in the PD group, 1.5%, than in the control group, 0.4% (*P* < 0.001). The incidence of periprosthetic mechanical complications was greater in the PD group, 1.1%, than in the control group, 0.6% (*P* = 0.024). The cogwheel rigidity and unpredictable spasticity of hamstrings after the anesthesia is worn off, preoperative flexion contracture needing more soft-tissue releases, can predispose to the posterior dislocation of the knee prosthesis.^11^ Using either cruciate retaining or semi or fully constrained prosthesis is recommended to avoid this catastrophic complication. Because the persons suffering from PD will be relatively less active, wear and other long-term concerns of the fully constrained prosthesis are minimized.^9^ Treating preoperative flexion contracture, with physical therapy serial casting, is recommended.^12^ However, a generalized statement cannot be made regarding the implant of choice in patients with PD presenting in various stages of disease and various levels of activity.

The bundled payment model for total joint arthroplasty was introduced in 2012 to decrease the highly variable cost and quality associated with the fee-for-service payment model. The results of this model are promising; however, this can hinder access to care of patients with more comorbidities, especially when the cost of the care exceeds the reimbursement per episode of care.^13^ The average total incurred charges for the PD group were higher, with a mean of $67,581.58 and a SD of $44,554.64, than that for the control group, with a mean of $46,493.28 and a SD of $71,072.26 (*P* < 0.001). The need for revision implants to avoid mechanical complications, prolonged surgical time for exposure, increased incidence of blood transfusions, and involving multiple specialist teams and personnel can add up to the increased cost per episode care in this subgroup.

The incidence of pneumonia was greater in the PD group, 0.3%, than in the unmatched control group, 0.1% (*P* = 0.014). We think most of this could be secondary to aspiration pneumonia. Aspiration pneumonia is a cause for concern in patients with PD. This complication may be due to oropharyngeal dysphagia, decreased cough reflex, and oral dysfunction. These symptoms might exaggerate after narcotics administration. However, we did not find an increased risk of pneumonia in the matched cohort.^14^

There is no statistically significant difference in the other immediate postoperative complications, such as mortality, acute myocardial infarction, pulmonary embolism, deep vein thrombosis, periprosthetic fracture, infection, and wound dehiscence, in the PD group, both matched and unmatched cohorts.

### Limitations

Our study has limitations that are inherent to most database studies. Most databases retrospectively collect data and report them using current procedural terminology (CPT) or ICD codes. This process leads to the loss of granularity of the information and unavailability of important data such as laboratory values and functional scores. In addition, the accuracy of the data collected depends on the ability of the database to translate the clinical information correctly, subjecting the database to reporting errors and ICD coding errors. Another limitation was that only 20% of the hospitals in the United States were represented in the NIS database. However, considering the large size of the database and extensive reliance on complex ICD codes, it is safe to assume that this database is a fair representation of the population undergoing TAA at the national level. These data pertain only to the inpatient database and cannot completely estimate short-term or long-term clinical and functional outcomes of TKA in PD. Some of the patients could be discharged to rehabilitation facilities versus home, which can technically skew the real COC and LOS data. PD is a progressive neurodegenerative disease and has multiple stages. The patient's outcome can depend on which stage the patient presents, and this information cannot be generalized to patients with all stages of PD. Cost of care in the United States is multifactorial and mostly depends on the contracts with existing implant companies. Most of these contracts are not uniform throughout the United States. However, we used a large database yielding a cohort of 3082 patients with PD, which is difficult to achieve with any single-center study. We also matched the patients with PD to non-PD patients, thereby increasing the strength of the findings. We have analyzed immediate surgical complications, unlike some other database studies that reported all complications as one group. With outpatient total joint arthroplasty and bundled payment, it is important to consider immediate complications, and surgeons must carefully select patients accordingly.

## Conclusion

This study concludes that there has been no notable increase in the incidence of PD in patients undergoing TKA since the last decade. There is an increased risk of ABLA, risks of increased blood transfusions, increased risk of prosthetic dislocations, and higher COC in a matched cohort and unmatched cohort. There is a notable increase in LOS and early postoperative pneumonia incidence in the unmatched cohort, but this trend is not observed in the matched cohort. There is no statistically increased risk of immediate early postoperative (inpatient) mortality, pulmonary embolism, DVT, periprosthetic fracture, infection, and wound dehiscence.
